# Neuropilin-1 modulates the 3D invasive properties of glioblastoma stem-like cells

**DOI:** 10.3389/fcell.2022.981583

**Published:** 2022-09-20

**Authors:** Mathilde Kerhervé, Sara Rosińska, Kilian Trillet, Alya Zeinaty, Magalie Feyeux, Steven Nedellec, Julie Gavard

**Affiliations:** ^1^ Team SOAP, CRCI2NA, Nantes Université, Inserm, CNRS, Université D’Angers, Nantes, France; ^2^ Equipe Labellisée Ligue Contre le Cancer, Nantes, France; ^3^ Nantes Université, CHU Nantes, CNRS, Inserm, BioCore, US16, SFR Bonamy, Nantes, France; ^4^ Institut de Cancérologie de L’Ouest (ICO), Angers, France

**Keywords:** adhesion, collagen, glioma, integrin, invasion, migration

## Abstract

Glioblastoma multiforme (GBM) is a rare, yet devastating, primary brain tumor in adults. Current treatments remain generally ineffective and GBM almost invariably recurs, resulting in median survival of 15 months. This high malignancy sources notably from the resilience and invasive capabilities of tumor cells. Within GBM, exists a population of self-sustaining transformed cells with stem-like properties (GSCs), which are thought to be responsible for tumor initiation, growth, and invasion, as well as recurrence. In the tumor microenvironment, GSCs might be found in the vicinity of brain endothelial cells, which provide a protective habitat. Likewise, these resistant, quiescent GSCs may accumulate in hypoxic zones, away from the perivascular niche, or travel towards the healthy brain parenchyma, by eminently co-opting neuro-vascular tracks. Herein, we established an *ex vivo* model to explore GSC invasive behavior. We found that patient-derived cells massively invade the collagen matrix. In addition, we described that the glycoprotein Neuropilin-1 (NRP1) contributes to GSC spreading and invasion. Indeed, both RNA interference-mediated silencing and CRISPR-mediated gene editing deletion of *NRP1* strongly impaired the 3D invasive properties of patient-derived GSCs and their close localization to the brain blood vessels. Of note, other typical features of GSCs, such as expansion and self-renewal were maintained. From a mechanistic standpoint, this biological effect might rely on the expression of the β3 subunit integrin cell-extracellular matrix adhesive receptor. Our data, therefore, propose a reliable approach to explore invasive properties of patient glioma cells *ex vivo* and identify NRP1 as a mediator in this malignant process.

## Introduction

Glioblastoma multiforme (GBM) is adults’ most common and serious form of primary brain tumor. Despite standard care management, including surgery combined with the so-called Stupp protocol (Stupp et al., 2005), i.e., radiotherapy and chemotherapy with temozolomide (TMZ), relapse is virtually inevitable and fatal. The failure of the conventional therapies can be in part explained by glioblastoma stem-like cells (GSCs) (Lathia et al., 2015). Such ill cells are not only capable of initiating, maintaining, and repopulating the tumor mass after treatments but are also remarkably invasive and able to disseminate in the central nervous system (Winkler et al., 2009). While scarcely metastasizing out of the brain, GBM cells can practically travel and spread mm-range distance. Distal areas can be colonized through the brain parenchyma, white matter tracts, leptomeningeal space, and perivascular routes, in the so-called “secondary structures of Scherer” (Cuddapah et al., 2014).

From a molecular standpoint, GSCs coexist in different molecular states recapitulating their origin and fate, resulting in a rapidly evolving, heterogeneous tumor cell population (Verhaak et al., 2010; Darmanis et al., 2017; Neftel et al., 2019). This plasticity strongly relies on their ability to integrate and exploit the biomechanical cues emanating from their microenvironment (Barnes et al., 2017). Notably, the perivascular habitat might govern GSC fate (Hambardzumyan and Bergers, 2015). This proximity between GSCs and the brain endothelial mats has been unveiled *in situ* and in experimental models, in which brain endothelial cell-emanating factors sustain GSC stem-like states (Calabrese et al., 2007; Galan-Moya et al., 2011; Krusche et al., 2016; Darmanis et al., 2017; Harford-Wright et al., 2017). Paralleling the adult neurogenic niche, the gliovascular unit includes endothelial cells, pericytes, astrocytes, and myeloid cells (Cuddapah et al., 2014). This vascular niche, therefore, forms a privileged entity to serve and protect bidirectional dialog between GSCs and endothelial cells (Watkins et al., 2014; Harford-Wright et al., 2017). Likewise, GSCs locate within alternate, defined territories, which equally orchestrate their identity and outcome (Hambardzumyan and Bergers, 2015). GSCs might also reside in hypoxic and invasive niches. The latest might bolster their migratory and invasive behavior along the vascular structures (Darmanis et al., 2017; Mathivet et al., 2017; Griveau et al., 2018; Alieva et al., 2019; Daubon et al., 2019). Accordingly, GBM cells barely disseminate using intravasation within the bloodstream, unlike most peripheral tumor cells that misuse vessel and lymphatic circulation routes (Rosińska and Gavard, 2021). Paralleling the movement of the progenitors and immature cells in the neural stem niche, GSCs co-opt blood vessels to migrate and expand (Cuddapah et al., 2014; Krusche et al., 2016; Daubon et al., 2019; Seano and Jain, 2020). In this regard, GSCs display an elevated invasive potential compared to non-stem, differentiated tumor cells (Pencheva et al., 2017).

GBM invasion requires fine, dynamic coordination of interactions between tumor cells and brain extracellular matrix (ECM). The ECM composition and overall architecture actively assist GSC migration. A quantitative and qualitative variety of extracellular proteins, including hyaluronan, vitronectin, collagens, and tenascin contribute to the adhesive and infiltrative behavior (Cosset et al., 2012; Hatoum et al., 2019). Because integrins (ITG) bridge ECM proteins to the actin cytoskeleton and intracellular signaling, they are a cornerstone for cell migration and invasion (Desgrosellier and Cheresh, 2010). These cell-surface receptors formed by the combination of α- (18) and β-subunits (8) can assemble a selective repertoire toward ECM ligands. Even if integrins are linked to GBM invasion and malignancy, their therapeutic targeting remains jeopardized (Malric et al., 2017). Hence, there is a need to elucidate new “druggable” modulators involved in GSC spreading. While detailed mechanisms were characterized in *ex vivo* models using mainly 2D “flat” culture conditions, recent efforts were made toward improved models that integrates both the 3D structure and composition of the tumor stroma (Pencheva et al., 2017; Koh et al., 2018; Mair et al., 2018). These experimental settings use cell lines, induced-pluripotent cells, and patient material applied to decellularized matrices, organotypic brain slices, matrix hydrogels, and fabricated scaffolds (Brassard-Jollive et al., 2020). Here, we revisited a 3D invasion assay in serum-free hydrogel matrix using patient cells that can be manipulated and imaged to evaluate their infiltrative and growth behavior. We highlighted the role of neuropilin-1 (NRP1) as a co-dependent factor of β-integrins in the invasive properties of patient-derived GSCs. In the neural and vascular systems, NRP1 acts a co-receptor for VEGF/VEGFR and Class III Semaphorins (Raimondi et al., 2016). Alternate roles for NRP1 in TGFβ signaling (Kwiatkowski et al., 2017; Roy et al., 2017) and SARS-cov-2 infection (Cantuti-Castelvetri et al., 2020; Daly et al., 2020) have been reported. Here, we found that genetically targeting *NRP1*, through siRNA knockdown and CRISPR/Cas9-mediated gene editing, inhibited GBM patient cells’ 3D invasion and segregated them away from brain vasculature. Moreover, we established that *ITGB3* silencing phenocopied *NRP1* deficiency. Collectively, our data support the concept that GSCs are intrinsically, highly infiltrative cells that pirated their vascular environment.

## Methods

### Ethics statement

Informed consent was obtained from all patients prior to sample collection for diagnostic purposes. This study was reviewed and approved by the institutional review boards of Sainte Anne Hospital, Paris, France, and Laennec Hospital, Nantes, France, and performed per the Helsinki Protocol. Animal procedures were conducted in agreement with the European Convention for the Protection of Vertebrate Animals used for Experimental and other Scientific Purposes (ETS 123) and approved by the French Government (APAFIS#24400-2020022713064016.v2). 7–8 weeks-old male and female C57BL/6 mice (Janvier Labs) were housed in specific pathogen-free conditions.

### Cell culture and siRNA

Two different patient-derived glioblastoma stem-like cells from patient #1 (GSC1, mesenchymal, 68-year-old male) and patient #9 (GSC9, classical, 68-year-old female), were isolated and grown as spheres in mitogen-defined serum-free medium (NS34, DMEM/F12 with B27, G5, and N2 supplements, GlutaMAX and antibiotics; Life Technologies, Harford-Wright et al., 2017). Stealth non-silencing control duplexes (low-GC 12935200, Life Technologies) and small interfering RNA duplexes targeting human *NRP1* (a mixture of HSS113022, HSS189594, HSS189595 in GSC1 and HSS113022 in GSC9, Life Technologies), *ITGB3* (5′-GCU​CAU​CUG​GAA​ACU​CCU​CAU​CAC​CTT-3′), and *GFP* (#EHUEGFP, Sigma) were transfected using RNAiMAX lipofectamine (Life Technologies). Additionally, GSC9 was transduced with GFP and luciferase (pLNT-LucF/pFG12-eGFP mixture plasmid, a gift of Valérie Trichet, Nantes Universite, France).

### Antibodies and reagents

The following primary antibodies were used for western blot (dilution 1:1000): GAPDH (SC-25778), ITGB1 (SC-365679), ITGB5 (SC-398214), ITGB7 (SC-515397), ITGB8 (SC-514150) from Santa Cruz Biotechnology, and ITGB3 (4702S, Cell Signaling Technologies). HRP-conjugated secondary antibodies (anti-rabbit, anti-mouse IgG1, IgG2a, and IgG2b) were purchased from Southern Biotech and used at 1:5000. APC-conjugated antibodies were used for flow cytometry: NRP1 (FAB3870A, R and D Systems, 1:20), ITGB1 (cat#559883, BD, 1:20), and ITGB3 (17-0619-42, Invitrogen, 1:20). Fasudil (Y_27632_, 50 nM) was from Sigma-Aldrich.

### CRISPR/Cas9 gene editing

Selected single guide RNA (sgRNA: 5′-CTT​CAA​CCC​TCA​CTT​CGA​TT-3′) targeting human *NRP1* was cloned into a lentiviral lentiCRISPRv2 backbone (GeCKO, ZhangLab), as described (Trillet et al., 2021). Puromycin-selected cells were single-cloned *via* NRP1 negative sorting (AriaIII, BD, Cytocell, SFR Francois Bonamy, Nantes). One bi-allelic KO clone was further selected after genomic DNA sequencing.

### The cancer genome atlas analysis

TCGA was explored *via* the Gliovis platform (http://gliovis.bioinfo.cnio.es/) (Bowman et al., 2017). RNAseq databases were used to interrogate clinical information associated with the expression of *ITGB1*, *NRP1*, and *NRP2*. The medium cutoff was applied, and all subtypes of primary GBM were included.

### Flow cytometry

Surface staining was performed on living cells by incubating antibodies for 1 h at 4°C. Flow cytometry analysis was performed on FACSCalibur (Cytocell, SFR Francois Bonamy, Nantes) and processed using FlowJo software (BD, version X).

### Western-blot

Cells were lysed in RIPA lysis buffer (25 mM Tris-HCl pH 7.4, 150 mM NaCl, 0.1% SDS, 0.5% Na-Deoxycholate, 1% NP-40, 1 mM EDTA, Pierce protease inhibitor) for 30 min on ice and cleared by centrifugation (10,000 g, 10 min, 4°C). 10 μg of post-nuclei supernatants were resolved by SDS-PAGE and transferred to nitrocellulose membranes. Signals were revealed using chemiluminescent HRP substrate (Merck) and visualized using Fusion imaging system (Vilber-Lourmart).

### RT-qPCR

Equal amounts of RNA (RNeasy, Qiagen) were reverse-transcribed (Maxima Reverse Transcriptase, Life Technologies), and 50 ng of cDNA was amplified (PerfeCTA SYBR Green SuperMix Low ROX, QuantaBio). Data were analyzed by the 2-d dCt methods and normalized with the housekeeping genes *ACTB* and *HPRT1*. The following primers (human targets) were used: *ACTB* forward 5′-GGA​CTT​CGA​GCA​AGA​GAT​GG-3′; *ACTB* reverse 5′-AGC​ACT​GTG​TTG​GCG​TAC​AG-3′; *HPRT1* forward 5′-TGA​CAC​TGG​CAA​AAC​AAT​GCA-3′; *HPRT1* reverse 5′-GGT​CCT​TTT​CAC​CAG​CAA​GCT-3′; *NRP1* forward 5′-ATC​ACG​TGC​AGC​TCA​AGT​GG-3′, *NRP1* reverse 5′-TCA​TGC​AGT​GGG​CAG​AGT​TC-3’; *ITGB1* forward 5′-CCT​ACT​TCT​GCA​CGA​TGT​GAT​G-3’; *ITGB1* reverse 5′-CCT​TTG​CTA​CGG​TTG​GTT​ACA​TT-3; *ITGB2* forward 5′-TTC​GGG​TCC​TTC​GTG​GAC​A-3’; *ITGB2* reverse 5′-ACT​GGT​TGG​AGT​TGT​TGG​TC-3’; *ITGB3* forward 5′-GTG​ACC​TGA​AGG​AGA​ATC​TGC-3’; *ITGB3* reverse 5′-CCG​GAG​TGC​AAT​CCT​CTG​G-3’; *ITGB4* forward 5′-CTC​CAC​CGA​GTC​AGC​CTT​C-3’; *ITGB4* reverse 5′-CGG​GTA​GTC​CTG​TGT​CCT​GTA-3’; *ITGB5* forward 5′-AAC​TCG​CGG​AGG​AGA​TGA​G-3’; *ITGB5* reverse 5′-GGT​GCC​GTG​TAG​GAG​AAA​GG-3’; *ITGB6* forward 5′-TCC​ATC​TGG​AGT​TGG​CGA​AAG-3’; *ITGB6* reverse 5′-TCT​GTC​TGC​CTA​CAC​TGA​GAG-3’; *ITGB7* forward 5′-CCA​TTC​AGC​TTT​CAC​CAT​GTG​C-3’; *ITGB7* reverse 5′-ACC​TTC​AGG​CGA​GTC​CAG​ATT-3’; *ITGB8* forward 5′-GTG​AAA​GTC​ATA​TCG​GAT​GGC​G-3’; *ITGB8* reverse 5′-GCT​ATC​AAG​AGC​GAG​ATG​AGA​CG-3’.

### Spheroid formation and 3D cell invasion assay

8,000 GSCs were seeded in a 96-well Ultra-Low Attachment plate (Corning) in 100 μl NS34 + 0.16% Methylcellulose (4000 cP, Sigma Aldrich), centrifuged (100 g, 20 min), and incubated for 2 days. Spheroids were embedded in 1 mg/ml collagen hydrogel (rat-tail collagen I, Corning, 1 M NaOH). Upon polymerization, 100 μl of NS34 was added. Alternatively, cells were placed into growth factor-reduced matrigel (Corning). Images were acquired at the indicated times with an inverted microscope (Nikon Tie), equipped with a cmos sensor using a 4x/0.13 objective (Nikon). All images were analyzed and quantified using Fiji ImageJ software (version 2.1.0, https://imagej.nih.gov/ij/). The invasive index was calculated as the ratio of the spheroid areas day2/day0.

### Cell viability assay

8,000 cells seeded in triplicate were treated as indicated for 3 days. Cell viability was measured using luminescent metabolic assay (CellTiterGlo, Promega) on a FluoStar Optima plate reader (BMG Biotech).

### Tumorsphere formation

Experiments were performed as described in (Harford-Wright et al., 2017). GSCs (60 cells/μl) were seeded in triplicate in NS34, dissociated manually from day 1–3, and analyzed on day 4. The number of tumorspheres was manually counted (single-blinded) in five random fields of view (fov) per well.

### Clearing and confocal analysis

Spheroids were collected, fixed (PBS-PFA 4%, 30 min), and permeabilized with PBS-Triton X-100 0.5%, BSA 3% (2 h). Alexa488-conjugated Phalloidin (1:300, Life Technologies) was added for 24 h. After PBS-Tween20 0.02% washes, spheroids were incubated for 24 h with TO-PRO-3 (1:1000, Life Technologies). Following washes, samples were cleared (2 days, 4°C, RapiClear 1.47, SunJin Lab). Images were acquired on a confocal microscope (Nikon A1rHD) using a 25x/1.05 silicon-immersion objective and reconstructed in 3D using the NIS-Element Software (Nikon).

### Migration on organotypic brain slices and image analysis

7–8 weeks old female and male mice were euthanized and brains were harvested. Brains were cut in 8–10 fragments on ice and embedded in a 5% low gelling temperature agarose at 37°C (Sigma-Aldrich). 250 µm thick brain slices (vibratome, Leica VT1000S) were transferred onto culture inserts (Merck Millipore) in NS34 culture media. 30,000 GreenCellTracker-stained GSCs (1:1000, 1 h, 37°C, Life Technologies) were added together with isolectin GS-IB4 dye (1:60, Life Technologies) and incubated for 1 h more at 37°C. Slices were washed, fixed (PBS-PFA 4%, 20 min), and imaged with a confocal microscope (Nikon A1r). A depth of up to 60 µm was acquired (20x/0.75 objective, 4–6 areas per slice). The distance between GSCs (green) and blood vessels (red) was quantified using Fiji ImageJ software (version 1.53q, Schindelin et al., 2012). Images were Z projected at max intensity for 2D analysis. GSCs were segmented using the StarDist plugins (Schmidt et al., 2018) and the distance map was calculated outside the thresholded vessels using Li method.

### Statistics

Data are representative of at least three independent experiments unless otherwise stated. Statistical analyses were performed using GraphPad Prism9 using an unpaired *t*-test (Student’s *t*-test), one-way analysis of variance (ANOVA), or two-way ANOVA. For each statistical test, a *p*-value of <0.05 was considered significant.

## Results

### Collagen-based 3D invasion of glioblastoma patient-derived cells

**FIGURE 1:**
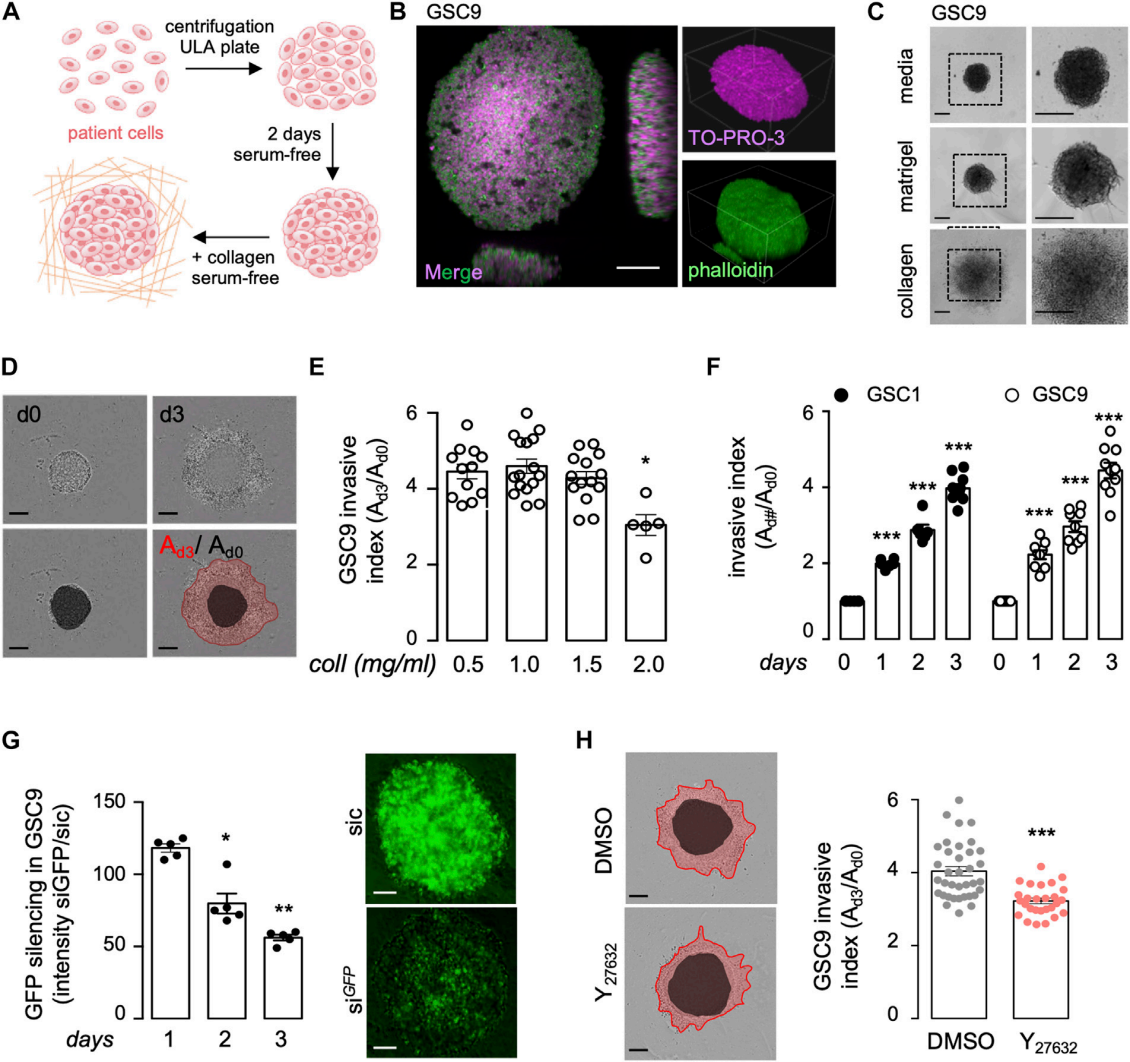
Collagen/integrin-based 3D invasion of glioblastoma patient-derived cells. **(A)** Schematic representation of the experimental design to assess spheroid formation and invasion. Briefly, 8,000 glioblastoma stem-like patient-derived cells (GSCs) were dissociated, centrifuged, and incubated for 2 days in a complete medium to allow spheroid formation. Spheroids were then embedded in collagen hydrogel to perform an invasion assay. **(B)** Spheroids were fixed, permeabilized, and clarified for further confocal microscopy of TO-PRO-3 (purple) and phalloidin (green) staining. Smaller insights x,y,z: 424 × 424 × 225 μm, Scale bar: 100 μm. **(C)** Representative bright-field images of spheroids floating in their complete serum-free media or embedded in matrigel and collagen for 2 days. Scale bars: 300 μm. **(D)** Typical bright-field images were acquired on days 0 (d0) and 3 (d3). The invasion index (Ad3/Ad0) was calculated as the ratio of the total area **(A)** for each spheroid at day 3 (red line) to day 0 (black circle), Scale bars: 300 μm. **(E)** 3D invasion assays of 8,000 GSC9 were performed in increasing concentrations of collagen (Coll, 0.5, 1.0, 1.5, and 2.0 mg/ml). Graph represents the mean ± s.e.m. of the invasive index on 5–16 spheroids from three independent experiments. **(F)** 3D invasion assays were performed with 8,000 GSC1 and GSC9 at the indicated time points. Graph represents the mean ± s.e.m. of the invasive index on 5–9 spheroids from three independent experiments. **(G)** GSC9 stably expressing GFP were transfected with non-silencing (sic) or GFP targeting duplexes RNA duplexes (si^GFP^). The knockdown efficiency was evaluated *via* GFP signal intensity at the indicated times. Graph represents the mean ± s.e.m. of the si^GFP^/sic ratio in five spheroids from two independent experiments. Scale bars: 100 μm. **(H)** Spheroids were embedded in 1 mg/ml collagen hydrogel and were treated with either vehicle (DMSO) or the ROCK inhibitor (Y_27632_, 50 mM) for 72 h. Graph represents the mean ± s.e.m. of the invasive index on 27–36 spheroids from three independent experiments. Representative bright-field images are shown. Scale bars: 300 μm. All panels are representative of at least three independent experiments unless otherwise stated, *t*-test and ANOVA, *p < 0.05, **p < 0.01, ***p < 0.001.

To study the infiltrative behavior of patient-derived glioblastoma cells (GSCs), we applied a two-step process that allowed 1) to control for spheroid size and growth upon centrifugation of isolated patient cells, and, 2) to examine invasion in a serum-free defined matrix (Figure 1A). First, confocal analysis of spheroids that were cleared and stained with TO-PRO-3 to mark nuclei and Phalloidin to illuminate actin structures confirmed the integrity of the engineered spheroids (Figure 1B). Next, collagen was preferred over commonly used matrigel, in which GSC invasion was prevented (Figure 1C). We estimated the invasion index by the ratio of the invasive area (as delimited with the red line), normalized to the area of the core (as identified with the black circle) (Figure 1D). Collagen concentrations ranging from 0.5 to 1.5 mg/ml provided effective support for the invasion, as opposed to concentrations above 2 mg/ml that strongly hampered it (Figure 1E), most likely due to alterations of the bio-physical properties of the as prepared hydrogels (Antoine et al., 2014). At the chosen medium 1 mg/ml collagen concentration, invasion index augmented over time (from day 1–3), and was visible in two patient-derived lines (namely mesenchymal GSC1 and classical GSC9) with comparable intrinsic invasive properties (Figure 1F). Finally, we evaluated whether these experimental settings can be challenged with genetic and pharmacological manipulation of patient cells. As a proof-of-concept, GFP can be efficiently knocked-down in stably GFP-expressing spheroids (Figure 1G). Likewise, the use of Y_27632_, a ROCK inhibitor also known as fasudil, a well-established inhibitor of cell motility (Basile et al., 2007), reduced the invasion index (Figure 1H). Thus, we developed a reliable method to assess patient cell invasion in collagen scaffolds.

### Neuropilin-1 expression emerges as a co-dependent factor of integrins in glioblastoma multiforme

**FIGURE 2:**
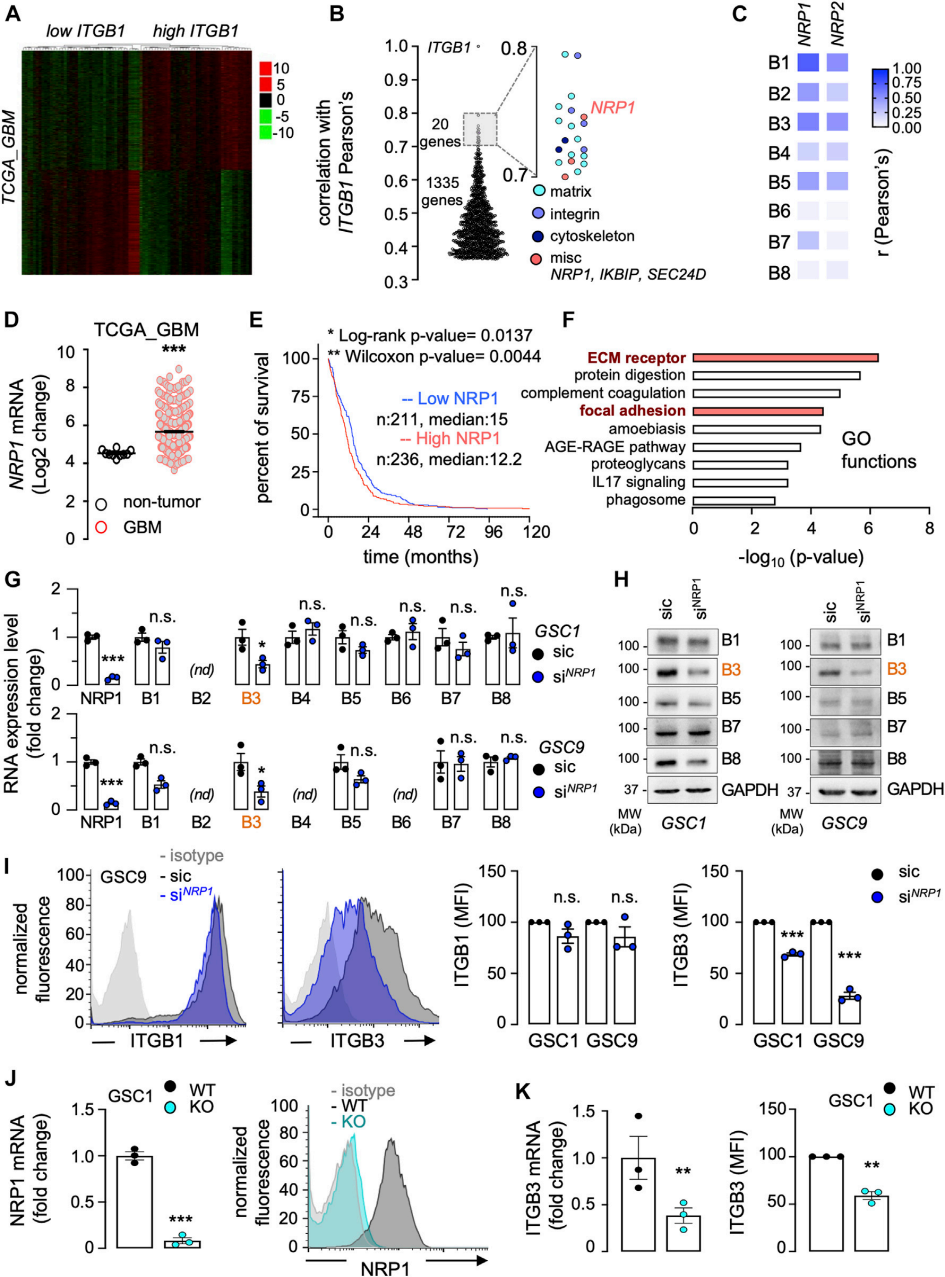
Neuropilin-1 expression emerges as a co-dependent factor of integrins in GBM. **(A–F)** The Cancer Genome Atlas (TCGA RNAseq dataset) was interrogated *via* the GlioVis platform to analyze differential gene expression in 528 GBM patients. Patients were clustered between low or high *ITGB1* mRNA expression (median cut-off). Representation of heatmap **(A)** and Pearson’s correlation factors **(B)** between each differentially expressed gene and *ITGB1*, with a focus on the ones with a positive correlation ranging from 0.7 to 0.8. Pearson’s correlation heatmap analysis of the association between *NRP1* and *NRP2* mRNA expression level and each *ITGB* subunit (B1 to B8) **(C)**. *NRP1* mRNA expression from non-tumor samples, as compared to GBM patient samples **(D)**. Kaplan-Meier survival curve (percent) for 528 GBM patients clustered between low or high *NRP1* mRNA level, using median cut-off **(E)**. Down and upregulated genes associated with *NRP1* were analyzed for their GO (gene ontology) functions. Each GO function is shown with its corresponding *p*-value (-log10) **(F)**. **(G–I)** Glioblastoma stem-like patient-derived cells (GSC1 and GSC9) were transfected with non-silencing (sic, black) and *NRP1* targeting duplexes (si^*NRP1*^, blue). The knockdown efficiency and the level of ITGB subunits (B1 to B8) was analyzed by RT-qPCR **(G)** and western-blot **(H)**. *ACTB* and *HRPT1* were used as housekeeping genes for normalization in **(G)** and GAPDH was used as a loading control **(H)**. *nd*, not detected. Cell surface flow cytometry analysis in GSC1 and GSC9 using the indicated antibodies for ITGB1 and ITGB3. Histograms present the mean fluorescence intensity (MFI) normalized to sic. Data are representative of three different experiments **(I)**. **(J)** CRISPR-Cas9 gene editing-mediated depletion of *NRP1* was evaluated in *NRP1* KO GSC1 (light blue) at the RNA and protein level, and compared to WT GSC1 (dark grey). qPCR analysis of *NRP1* mRNA is shown as the mean ± s.e.m. fold change from three independent experiments using *ACTB* and *HRPT1*, as housekeeping genes for normalization (left panel). Representative plots of flow cytometry analysis show the staining of immunoglobulin isotype control (light grey), WT (dark grey), and *NRP1* KO (light blue). **(K)** The level of ITGB3 was analyzed by RT-qPCR (left panel) and by cell surface flow cytometry (right panel). *ACTB* and *HRPT1* were used as housekeeping genes for normalization. All panels are representative of at least three independent experiments, *t*-test and ANOVA, *p < 0.05, **p < 0.01, ***p < 0.001.

Several studies suggest that integrins, notably α_v_β_3_ and those that encompass the β_1_ subunit, regulate glioblastoma cell invasion (Martin et al., 2012). To identify ITGB1-related genes whose expression might impact the disease outcome, we interrogated The Cancer Genome Atlas (TCGA) through the Gliovis platform (Bowman et al., 2017) (Figure 2A). A total of 2,438 genes were found differentially expressed (i.e., adjusted *p*-value ≤ 0.05) between high and low *ITGB1* expressed samples (median cutoff), among which 1,355 and 1,083 genes were positively and negatively associated, respectively (Figure 2A). A focus on the *ITGB1*-positive correlated genes highlighted three groups of 20 genes with a Pearson’s correlation r factor ranging between 0.7 and 0.8. Their GO functions were related to 1) matrix biology and remodeling (*COL1A1*, *COL1A2*, *COL3A1*, *COL4A1*, *FN1*, *FNDC3B*, *LAMC1*, *LOXL2*, *LUM*, and *SERPINH1*, please see the light-blue dots), 2) integrin sub-units (*ITGA1*, *ITGA4*, and *ITGA5*, please see the regular-blue dots), and 3) actin cytoskeleton and adhesion (*CD93*, *LMAN1*, *TES*, *TMP4*, please see the dark-blue dots) (Figure 2B). Of note, three miscellaneous genes namely *IKBIP, SEC24D*, and *NRP1* (please see the red dots)*,* emerged from this data mining*.* The glycoprotein neuropilin-1 (NRP1) was retained, as it was previously linked to integrin functions in the context of angiogenesis (Serini et al., 2008). Likewise, NRP1 has been involved in glioma cell migration processes, notably in 2D and chemotaxis models (Evans et al., 2011; Angom et al., 2020). To get further insights on the codependency between *NRP1* and *ITGB* subunits in glioblastoma, TCGA was again queried (Figure 2C). The expression level of *NRP1*, and to a lesser extent of *NRP2*, strongly correlated with the one of *ITGB1*, *ITGB3*, and *ITGB5* (Figure 2C). In agreement with previous studies (Angom et al., 2020), *NRP1* expression was over-represented in glioblastoma patients, as opposed to non-tumor brain tissue (Figure 2D). The Kaplan-Meier survival analysis on TCGA database that includes 320 primary GBM patients suggests that high *NRP1* expression (median cutoff) is of slight worsen prognosis (log-rank *p*-value 0.0204, Wilcoxon *p*-value 0.0072), with a noticeable difference in the earlier time points (Figure 2E). Paralleling the situation with endothelial cells (Serini et al., 2008), *NRP1*-dependent differentially expressed genes notably clustered in two biological functions related to integrin functions, namely “ECM receptor” and “focal adhesion” (Figure 2F). Alternate signatures also emerged and involved either dynamic adhesion and morphological remodeling processes (e.g., “protein digestion and absorption,” “proteoglycans in cancer”) or immune system and responses (e.g., “amoebiasis,” “complement and coagulation cascade,” “AGE-RAGE signaling pathway,” “IL17 signaling pathway,” “phagosome”) (Figure 2F). To next evaluate the relevance of the interconnection between ITGB and NRP1 in GSCs, *NRP1* was knocked-down with transient RNA interference in two patient-derived cells (Figure 2G). This significantly reduced the level of ITGB3, at the RNA and protein level, while sparing other tested ITGB subunits, among which ITGB1, in GSC1 and GSC9 (Figures 2G,H). Of note, B2 in GSC1 and B2, B4, and B6 in GSC9 integrin subunits were barely expressed. Further flow cytometry analysis confirmed that *NRP1* silencing caused a significant reduction in the surface staining of ITGB3, but not of the widely expressed ITGB1, in the two different patient-derived GSCs (Figure 2I). To reinforce our findings, a CRISPR-Cas9-based bi-allelic knockout of *NRP1* was designed and selected in GSC1 (Figure 2J). *NRP1* deletion resulted in a significant reduction of both RNA and protein surface staining of ITGB3, but not of ITGB1 (Figure 2K). Our data strengthened the concept that NRP1 is an integral part of the integrin network in GSCs.

### Neuropilin1 expression modulates patient-derived glioblastoma stem-like patient-derived cells 3D cell invasion

**FIGURE 3:**
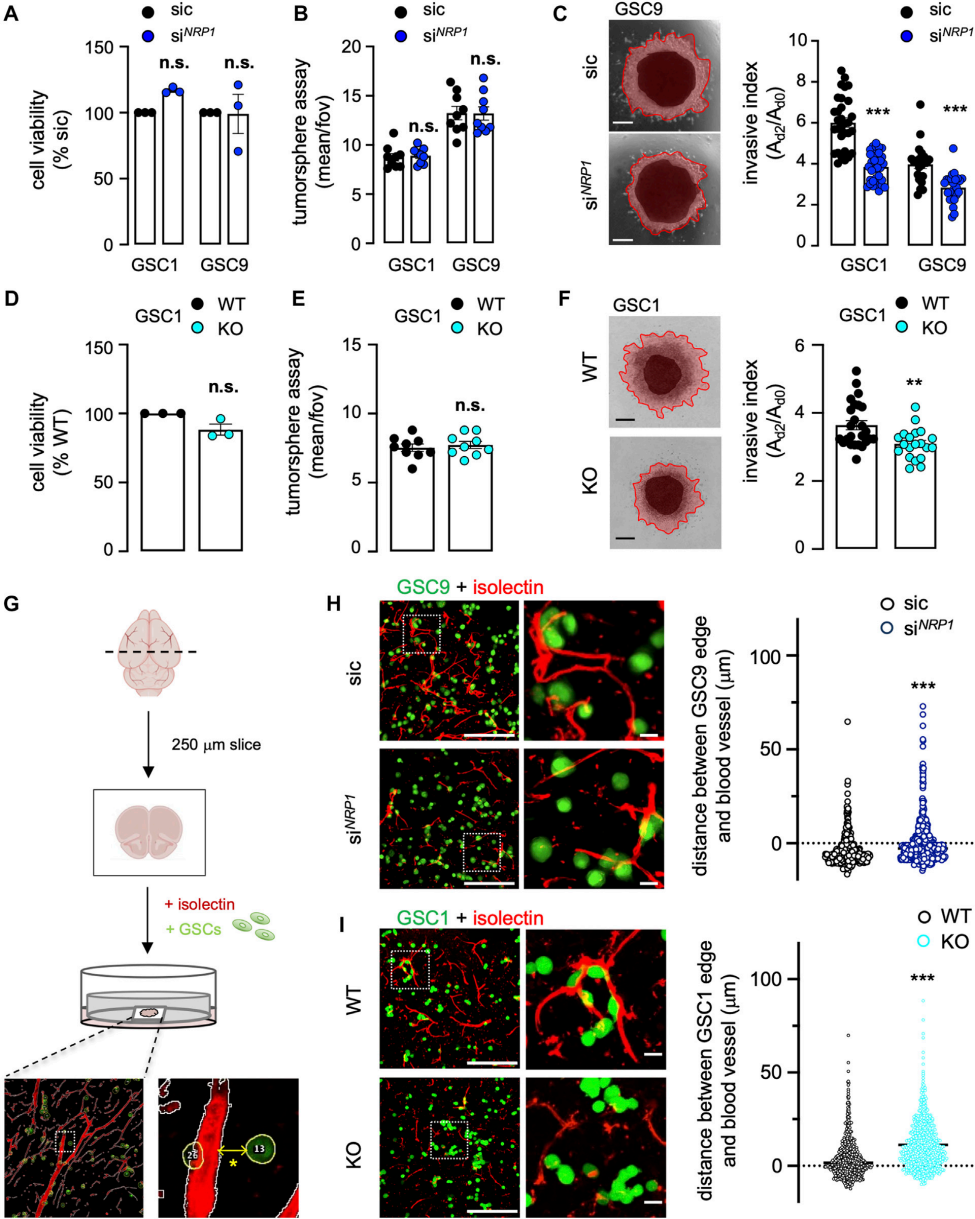
Neuropilin1 expression modulates patient-derived GSC 3D cell invasion. **(A–C)** Glioblastoma stem-like patient-derived cells (GSC1 and GSC9) were transfected with non-silencing (sic, black) and *NRP1* targeting duplexes (si^*NRP1*^, dark blue). Cell viability was measured using CellTiterGlo on day 3. Data were normalized to sic and are presented as the mean ± s.e.m. of three independent experiments in triplicate **(A)**. Tumorspheres per field of view (fov) were single-blinded and manually counted. Data are presented as the mean ± s.e.m. from three independent experiments in triplicate **(B)**. Representative images and quantification of invasion assay in collagen embedded-spheroids at day 3. Scale bars: 300 μm. Data are presented as the mean ± s.e.m of the invasive index on 22–33 spheroids from three independent experiments **(C)**. **(D–F)** Cell viability was measured using WT and *NRP1* KO GSC1 on day 3 **(D)**. Tumorspheres per field of view (fov) were single-blinded and manually counted **(E)**. Representative images and quantification of invasion assay of WT and *NRP1* KO GSC1 spheroids embedded in 1 mg/ml collagen hydrogel **(F)**. Data are presented as the mean ± s.e.m of 20–25 spheroids from three independent experiments. Scale bars: 300 μm. **(G)** Schematic representation of the experimental design for organotypic brain slice cultures, in which Alexa488-conjugated CellTracker-labeled GSCs (green) were added. TRITC-conjugated isolectin was used to illuminate blood vessels (red). A representative image for automatic distance quantification is shown below. The * denotes the distance between the edges of the CellTracker-labeled GSC and Isolectin-labeled vessel. **(H–I)** Representative confocal images of the co-culture between GSCs (green) and mouse brain. The distance between the edge of each GSC and the nearest blood vessel (red) was further quantified in non-silencing (sic, black) and *NRP1* targeting duplexes (si^*NRP1*^, dark blue) GSC9 **(H)**, and, WT (black) and *NRP1* KO GSC1 (light blue) **(I)**. Representative n > 427 of two independent experiments **(H)**, n > 770 from two independent experiments **(I)**. Scale bars: 200 μm, 20 μm. All panels are representative of at least three independent experiments unless otherwise stated, *t*-test and ANOVA, **p < 0.01, ***p < 0.001.

We next explored the functional impact of *NRP1* silencing on the invasive properties of patient-isolated GSCs. First, *NRP1* knockdown did not impact neither GSC viability nor their ability to form spheres in floating non-adherent conditions (Figures 3A,B). Strikingly, the invasion in the collagen-based 3D matrix was significantly reduced in *NRP1*-silenced GSCs (Figure 3C). Likewise, *NRP1* KO did not influence GSC viability and tumorsphere formation, yet resulted in the loss of invasion in 3D collagen scaffolds, therefore recapitulating the effect of the transient knockdown (Figures 3D–F). Because GSCs can invade the brain parenchyma by co-opting vessel routes on collagen tracks (Mancuso et al., 2006; Griveau et al., 2018; Rosińska and Gavard, 2021), we next deployed a model of organotypic brain slice culture. This system not only recapitulates the 3D brain architecture and stroma composition but also allows the tracking and visualization of fluorescent-labeled GSC behavior in interaction with isolectin-marked vasculature (Figure 3G). In such conditions, control GSC9 were mainly found in the proximity of vessels, largely overlapping (Figure 3H). Conversely, *NRP1* knocked-down cells were stochastically dispersed on the brain slices, as the mean distance between GSCs and the closest blood vessels was significantly heightened (Figure 3H). Similarly, while WT GSC1 were in close contact with vessels, *NRP1* KO cells positioned at distance, recapitulating the effects of the transient knockdown (Figure 3I). Our data thus highlighted the pivotal role of NRP1 in controlling the motility of GSCs, notably toward the host vasculature.

### *ITGB3* silencing recapitulates the NRP1 deficiency phenotype in patient-derived glioblastoma stem-like patient-derived cells

**FIGURE 4:**
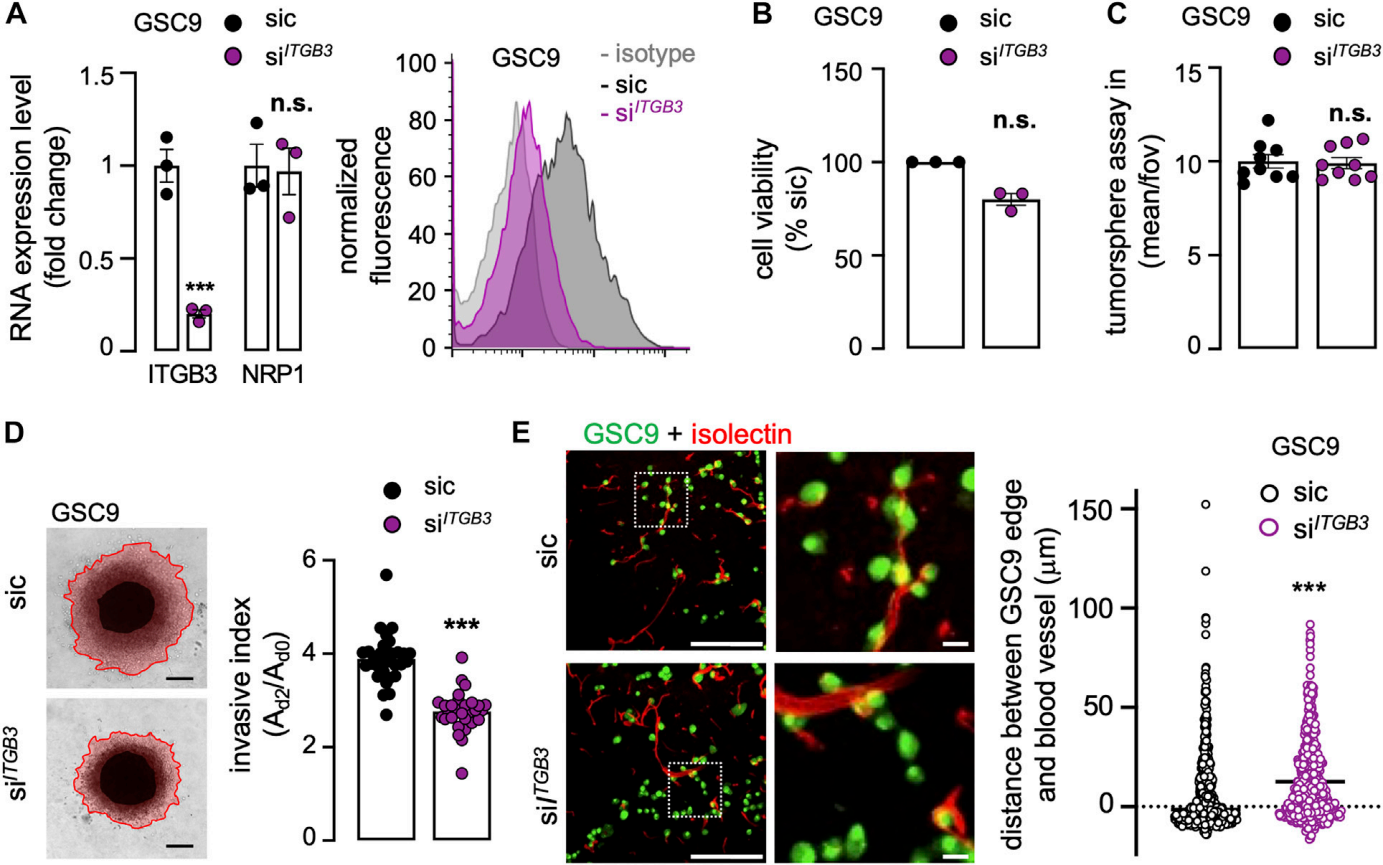
*ITGB3* silencing recapitulates the NRP1 deficiency phenotype in patient-derived GSCs. GSC9 were transfected with non-silencing (sic, black) and *ITGB3* targeting duplexes (si^*ITGB3*^, purple). **(A)** The knockdown efficiency was evaluated 72 h later at the RNA (left panel) and protein level (right panel). Alternatively, *NRP1* mRNA level was analyzed. qPCR analysis of *ITGB3* and *NRP1* mRNA is shown as the mean ± s.e.m. fold change from three independent experiments using *ACTB* and *HRPT1* as housekeeping genes for normalization (left panel). Representative plots of flow cytometry analysis show the staining of isotype control (light grey), sic (dark grey), and si^*ITGB3*^ (purple) (right panel). **(B)** Cell viability was measured using CellTiterGlo on day 3. Data were normalized to sic and are presented as the mean ± s.e.m. of three independent experiments in triplicate. **(C)** Tumorspheres per field of view (fov) were single-blinded and manually counted. Data are presented as the mean ± s.e.m. from three independent experiments in triplicate. **(B)** Representative images and quantification of invasion assay in collagen embedded-spheroids at day 3. Scale bars: 300 μm. Data are presented as the mean ± s.e.m of the invasive index on 29–31 spheroids from three independent experiments. **(E)** Representative confocal analysis of the co-culture between GSCs (green) and mouse brain. The distance between the edge of each GSC and the nearest blood vessel (red) was further quantified. Representative of two independent experiments (n > 522). Scale bars: 200 μm, 20 μm. All panels are representative of at least three independent experiments unless otherwise stated, *t*-test and ANOVA, **p < 0.01, ***p < 0.001.

To further explore the correlation between NRP1 and ITGB3 in GSC invasion, *ITGB3* was efficiently knocked-down using RNA interference (Figure 4A). It is noteworthy that *NRP1* expression was left intact in *ITGB3*-silenced cells, suggesting a non-reciprocal regulatory mechanism (Figure 4A). Again, viability and sphere formation were not significantly impacted by the knockdown of *ITGB3* (Figures 4B,C). Recapitulating *NRP1* deficiency, the 3D collagen-based invasion was drastically reduced upon *ITGB3* silencing (Figure 4D). In keeping with this, the mean distance of *ITGB3*-knockdown GSC9 from vessels increased (Figure 4E), like *NRP1* silencing phenotype. Collectively, these results suggest that *ITGB3* silencing mimics the *NRP1* inhibition-provoked phenotype, as invasiveness was repressed and GSCs were secluded from the host vasculature.

## Discussion

Glioblastoma is a deadly cancer for which treatments remain essentially palliative and did not evolve much since 2005. Besides late diagnosis, tumor infiltration in the brain parenchyma is one of the main pitfalls. In this study, NRP1 emerged as a cardinal receptor in orchestrating cell invasion, while linked to integrin functions.

Several reports indeed endorse a global over-representation of integrins in GBM, as returned notably from TCGA queries, as well as the noted increase of specific αβ heterodimers in clinical samples and experimental models (Desgrosellier and Cheresh, 2010; Malric et al., 2017). In keeping with this idea, the GBM connective tissue is strongly altered in terms of its biological composition and mechanical properties (Miroshnikova et al., 2016; Barnes et al., 2018). This extracellular matrix remodeling and stiffening sustain cell spreading and might be even more critical for the motility of the so-called mesenchymal subtype (Barnes et al., 2018). In this vein, integrins coordinate the dynamic interplay between invasive malignant cells and their supportive matrix soil (Desgrosellier and Cheresh, 2010). Extensive, yet disappointing, efforts have been implemented to translate promising data from experimental studies into clinical settings. α_v_β_3_ is the paradigm of such therapeutic opportunity, as this overexpressed integrin pair largely contributes to tumor angiogenesis and spreading (Demircioglu and Hodivala-Dilke, 2016). However, targeting α_v_β_3_ and/or α_v_β_5_ with the cilengitide compound is still debated and trialed (Weller et al., 2016).

Neuropilin-1 is an enigmatic glycoprotein transmembrane receptor, with no enzymatic activity that has been involved in many biological processes, including neural development and angiogenesis (Raimondi et al., 2016), as well as immune surveillance (Roy et al., 2017), and more recently SARS-cov-2 infection (Cantuti-Castelvetri et al., 2020; Daly et al., 2020). A large part of NRP1 functions is linked to its ability to bind and modulate the VEGF/VEGFR signaling pathway. In endothelial and tumor cells, NRP1 was shown to connect with β1 integrin subunits. Indeed, NRP1 contributes to governing integrin trafficking (internalization and vesicular delivery) (Valdembri et al., 2009). Herein, *NRP1* silencing is accompanied by a decrease in the level of ITGB3 subunit at the surface of GSCs, which is most likely provoked by a significant reduction of RNA and protein levels. The role of NRP1 might be specific to the β3 subunit, as the level of β1 was not overtly modified. Accordingly, *ITGB3* knockdown fully recapitulates the observed *NRP1* deficiency phenotype on invasion and brain colonization. The molecular mechanism is however not elucidated. For instance, the composition and architecture of the matrix might be a strong candidate, as it is thought that integrin heterodimers could exhibit differential interaction and dynamics depending on the presented matrices (e.g., fibronectin versus collagen-rich support), in addition to the relative level of expression and stability of each subunit and αβ heterodimers in GSCs. Future works are required to determine the underlying mechanisms of NRP1-driven ITGB3 downregulation.

The role of NRP1 in glioblastoma has been mainly explored in the context of endothelial cell biology, in response to VEGF, Class III Semaphorins, and integrins (Valdembri et al., 2009; Le Guelte et al., 2012). Furthermore, extensive work highlighted the functional action of NRP1 on glioblastoma expansion (Angom et al., 2020). By interfering with NRP1 expression, the authors demonstrated the importance of this glycoprotein, including stemness, migration, and resistance to treatments (Angom et al., 2020). While a large part of the NRP1-dependent phenotype can be recapitulated with VEGF knockdown, some might be disconnected and possibly linked to other signaling receptors, such as TGF-β (Glinka and Prud’homme, 2008; Kwiatkowski et al., 2017; Angom et al., 2020). Here, we established the autonomous function of NRP1 in modulating 3D invasion, independently of viability and sphere formation. Our results also highlighted the likely contribution of ITGB-based adhesion. How this effect is controlled in the presence of VEGF and/or TGF-β is underexplored. Nonetheless, our preliminary data suggest rather the modulation of the microtubule network in this context (data not shown). This is reminiscent of the proposed role of the tubulin cytoskeleton in the migrating behavior of glioblastoma cells, in response to biomechanical constraints (Monzo et al., 2021).

In this regard, malignant glioma cells, including GSCs can travel in the brain parenchyma and follow vessel tracks in a mechanism coined under the name of co-option (Rosińska and Gavard, 2021). Besides vascular co-option, seminal studies unveiled the role of neuronal connectivity and mimetism in brain tumor invasion (Venkatesh et al., 2019; Venkataramani et al., 2022). Because NRP1 orchestrates neuro-vascular patterning and navigation during development, it will be interesting to evaluate how much it contributes to neuronal-like brain infiltration. This dynamic, motile behavior has been recapitulated and documented in patient-derived samples and experimental models, including micropatterned lines, organotypic brain slice cultures, and intravital imaging in rodents (Caspani et al., 2014; Griveau et al., 2018; Alieva et al., 2019; Monzo et al., 2021; Venkataramani et al., 2022). This invasion mode involves a multi-step process that requires chemo-attraction to vessels, biophysical factors to maintain tumor cells at the proximity of the vasculature (including pericytes, endothelial cells, and surrounding matrix), and guidance to properly navigate. In this context, NRP1 is well-positioned to contribute to co-option. Our data suggest that NRP1 expression indeed governs the location of GSCs and their distance from blood vessels. This might be due to a lack of attraction toward the host vasculature, upon the release of vascular-emanating factors (Calabrese et al., 2007; Harford-Wright et al., 2017), and, cells, subsequently, stochastically migrate and invade the brain parenchyma. Earlier reports highlighted the role of EphrinB2 in the perivascular motion of GSCs (Krusche et al., 2016), and proposed blocking co-option as a therapeutic option. Likewise, both NRP1 and ITGB3 deficiency annihilated GSC positioning at the vicinity of vessels. How co-option contributes to glioblastoma spreading, response to treatments, and recurrence will be of paramount importance, in light the current therapeutic impasse in glioblastoma.

## Data Availability

The raw data supporting the conclusion of this article will be made available by the authors, without undue reservation.
